# Key features of applying unsupervised classification for monitoring spatiotemporal dynamics of landscape cover under conditions of an ecological disaster

**DOI:** 10.7717/peerj.21869

**Published:** 2026-10-27

**Authors:** Olena Lisovets, Hanna Tutova, Olha Kunakh, Olexander Zhukov

**Affiliations:** 1 Department of Biodiversity and Ecology, Oles Honchar Dnipro National University, Dnipro, Dnipro Region, Ukraine; 2 Department of Department of Botany, Ecology and Gardening, Bogdan Khmelnitsky Melitopol State Pedagogical University, Zaporizhzhia, Zaporizhzhia Region, Ukraine

**Keywords:** Sentinel-2, Spectral indices, Hierarchical clustering, Sequential principal component analysis (PCA), Floodplain ecosystems, Post-disaster succession, Land-cover dynamics, Habitat mapping, Kakhovka dam failure

## Abstract

The destruction of the Kakhovka Hydroelectric Power Plant dam in June 2023 triggered a rapid drawdown of the Kakhovka Reservoir and initiated a long-term reorganisation of the Dnipro River floodplain. Such post-disaster landscapes are characterised by the emergence of novel and transitional surface types, whose spectral properties and trajectories are difficult to anticipate, thereby limiting the use of supervised classification. The spatiotemporal dynamics of land cover (surface types) in the floodplain ecosystems of Khortytsia Island, Ukraine, were assessed using an unsupervised remote-sensing framework. A Sentinel-2 time series was analysed using a multi-index approach. Unsupervised classification was implemented *via* hierarchical clustering with objective selection of the number of clusters. A sequential ordination strategy was applied: initial clustering was conducted in the space of the first two principal components derived from principal component analysis, followed by residual principal component analysis after removal of the dominant components by regression to emphasise ecologically meaningful secondary variation, such as moisture and substrate signals. Cluster maps were optionally extrapolated to full rasters using linear discriminant analysis in the combined principal component analysis (PCA) and residual-PCA feature space. Clusters were interpreted through an expert crosswalk to the European Nature Information System habitat typology, supported by index profiles, spatial context, and seasonal behaviour. The discriminatory value of indices was also quantified at both the cluster level using standardised effect sizes and the global level using normalised importance weights. The surface-type system became progressively more differentiated throughout the growing season, evolving from a minimal early-season set to a more diverse assemblage of surface and habitat types by late season. This progression reflects rapid post-disaster succession and shoreline reconfiguration. Secondary components gained importance during peak vegetation development, indicating the increasing relevance of edaphic and moisture-related gradients in differentiating cover types when canopy biomass obscured non-vegetated signals. The ranking of informative indices shifted seasonally, from wetness and water sensitivity in late winter to early spring, to greenness and productivity in late spring and summer, and finally to substrate exposure and degradation signals in autumn. Unsupervised, sequential PCA-based clustering offers a robust framework for monitoring post-disaster floodplain transformations, as it accommodates novel classes, preserves subtle moisture and substrate signals, and supports interpretable temporal ‘genealogies’ of land-cover types. The results also indicate that relying solely on vegetation indices is insufficient for accurately mapping rapidly changing floodplain habitats following catastrophic disturbances.

## Introduction

Assessing the spatiotemporal dynamics of land-cover and habitat types using remote sensing data is a key prerequisite for effective biodiversity management and conservation (Timmermans & Daniel Kissling, 2023). Changes in land-cover structure determine resource availability, habitat connectivity, and population viability across spatial scales ranging from local microhabitats to regional ecological networks (Savary et al., 2024). Static land-use or habitat maps are insufficient for informed decision-making. Instead, it is necessary to track trajectories of transitions between land-cover and habitat classes, rates of change, hotspots of loss and recovery, and areas of high instability where risks to species and communities increase most rapidly (Pickens et al., 2025). This approach strengthens the evidence base for planning by supporting the selection of priority areas for protection and restoration, as well as the optimisation of buffer zones, ecological corridors, and the spatial allocation of measures that reduce anthropogenic pressure (Baker et al., 2025). Habitat types are directly linked to ecosystem functioning and to the suite of species’ functional traits (Lőrincz et al., 2025). Analysing their spatiotemporal dynamics enables a move beyond area-based descriptions towards assessments of habitat quality and the capacity to sustain key ecosystem services (Xu et al., 2024). Integrating spatiotemporal monitoring of land cover and habitats with data on species diversity, conservation status, and pressure factors facilitates earlier detection of negative trends, reduces uncertainty in forecasts, and increases the effectiveness of conservation strategies under climatic and socio-economic change (Antonelli, Dhanjal-Adams & Silvestro, 2023; Kass et al., 2024; Wang et al., 2025). This is especially important in the context of catastrophic transformations.

The use of remote sensing Earth observation data represents one of the most rapid and reproducible approaches for documenting the extent of disturbance, rates of degradation, and trajectories of ecosystem recovery during catastrophic events (Liu, Wang & Liu, 2025). In the case of ecosystem-scale catastrophic events such as wildfires, floods, droughts, windthrows, industrial accidents, hydraulic infrastructure failures, and war-related damage, an index-based approach facilitates the rapid mapping of impact zones and gradients of disturbance intensity (Pulvirenti, Squicciarino & Fiori, 2020). Spectral vegetation indices reflect the condition of photosynthetically active biomass and disruptions to phenological cycles (Zeng et al., 2022). Moisture and water indices are sensitive to inundation, shoreline shifts, drying, and floodplain reconfiguration (Irvine, de Mello & Mollick, 2026). Fire-related indices quantify burn severity and post-fire succession (Anees et al., 2025). Bare-soil and built-up indices indicate erosion processes, substrate exposure, and the expansion of artificial surfaces (Qi et al., 2024). Methodologically, the most informative framework is time-series analysis, which integrates pre-event baselines, event-related disturbance peaks, and post-event trends through change-detection techniques (Pasquarella et al., 2022; Nykytiuk, Kravchenko & Komorna, 2025). Spectral indices derived from remote sensing provide a standardised, spatiotemporally comparable toolkit for the quantitative assessment of catastrophic landscape transformations (Pulvirenti, Squicciarino & Fiori, 2020). Employing these indices as predictors in land-cover classification, and combining the resulting products with risk models and ecological indicators, enables a transition from damage mapping to evidence-based planning of restoration actions and conservation priorities.

Land-cover classification based on Earth remote sensing observation data can be conducted using either supervised or unsupervised approaches (Sharma et al., 2024). In conservation and restoration applications, achieving high accuracy in discriminating classes is critical, particularly for spectrally similar and spatially heterogeneous mosaics (Wen et al., 2025). Therefore, it is essential to incorporate predictors that reduce interpretative ambiguity and enhance the temporal robustness of results (Arfa & Minaei, 2024). Phenology captures regular seasonal changes in vegetation condition, thereby providing an additional temporal dimension that improves the separation of spectrally similar land-cover and habitat classes in supervised classification (Surasinghe, Singh & Smart, 2025). The utilisation of multi-date imagery or time series enables models to account for the shape of seasonal trajectories of vegetation indices, thus reducing the risk of misclassification (Jia et al., 2014). A more concise alternative is to derive phenological metrics and employ them as predictors in statistical procedures (Nguyen & Henebry, 2019). In the event of catastrophic disturbances, phenological anomalies assist in distinguishing short-term spectral artefacts from persistent disturbances and recovery processes, rendering change maps more interpretable for conservation planning (Labenski et al., 2024). However, supervised classification has fundamental limitations under catastrophic transformations, when class stability and the ‘normative’ character of training reference data are disrupted (Hertel et al., 2025). Following catastrophic events, traditional training categories become conditional, as transitional, mosaic, and rapidly changing states emerge in place of familiar land-cover types and do not always conform to the pre-event legend (Bao et al., 2024). These conditions elevate the risk of propagating reference-label errors across the mapped area, resulting in increased label noise, while simultaneously reducing the generalisation capacity of models trained on pre-catastrophe examples (Pelletier et al., 2017). Consequently, the focus of analysis increasingly shifts from changes in the area of individual classes to the diversity of newly formed habitat conditions, which reflect gradients of disturbance intensity and recovery trajectories (Bao et al., 2024). This, in turn, necessitates broader application of unsupervised classification approaches.

With the shift towards unsupervised classification, phenology ceases to be merely a methodological tool and becomes a distinct subject of study, as seasonal dynamics provide a crucial basis for interpreting the extracted clusters and land-cover states (Liu et al., 2026). Under a phenology-informed clustering approach, it is essential to distinguish genuinely catastrophic transformations from natural phenological variability and long-term phenological trends that may mimic land-cover change in spectral characteristics (Verbesselt et al., 2010a). The analysis should be based on comparing time series against a pre-event baseline, quantifying anomalies relative to quasi-stationary conditions, and identifying breaks in trajectories that cannot be explained by seasonality (Verbesselt et al., 2010b). This approach enables spatial patterns to be correctly interpreted as either disturbance-driven change or normal seasonal dynamics, thereby reducing the risk of false change detection.

Thus, the aim of this study is to investigate the spatiotemporal dynamics of land-cover types under conditions of an ecological disaster, using the floodplain ecosystems of Khortytsia Island (Ukraine) as a case study. These ecosystems were affected by the consequences of the destruction of the Kakhovka Reservoir. The objectives of the study are to: (1) develop and apply a sequential framework for ordination and unsupervised clustering to delineate land-cover types and their temporal transitions, and to evaluate the factors influencing the performance of unsupervised classification; (2) quantify the information value of spectral indices for differentiating spatial clusters; (3) analyse disaster-driven changes in landscape cover in the context of phenological dynamics across the growing season, including the emergence of new and transitional surface types and the redistribution of areas of key habitats; and (4) interpret the derived clusters in terms of habitat types by cross-referencing them with EUNIS, describing their ecological significance and trajectories of post-disaster transformation.

## Materials and Methods

### Overview of the study area and a brief account of the 2023 Kakhovka reservoir disaster

Khortytsia Island in Ukraine served as the case-study area for examining land-cover changes after the Kakhovka disaster (Lisovets et al., 2025). Khortytsia is the largest island in the Dnipro River, located near the city of Zaporizhzhia, downstream of the Dnipro Hydropower Plant (DniproHES). The island of Khortytsia, the largest island in the Dnipro River, is located in the vicinity of the city of Zaporizhzhia, downstream of the Dnipro Hydropower Plant (DniproHES). The island’s topography is characterised by an elongated orientation, extending from the northwest to the southeast, with a length of approximately 12 km and a width ranging from 1.6 to 2.7 km. Prior to the Kakhovka dam failure, the island’s total area was estimated to be approximately 2,359 hectares. Khortytsia is located within the northern part of the former Kakhovka Reservoir’s water area and its floodplain–riparian zone. Field surveys were conducted on the island during the years 2024–2025. Further comprehensive details pertaining to the spatial context, in addition to the topographic and ecological characteristics of the study area, are elucidated in our preceding publications (Tutova et al., 2026c, 2026d).

On 6 June 2023, the dam of the Kakhovka Hydroelectric Power Plant was destroyed in an explosion, causing a rapid drawdown of the Kakhovka Reservoir and a substantial decrease in water levels across the Dnipro River system. At the Nikopol hydrological gauge, the river level fell by approximately 8 m (Gleick, Vyshnevskyi & Shevchuk, 2023). The collapse of the dam led to prolonged reorganisation of floodplain conditions in the Dnipro system. The main manifestations of this process included shifts in hydrology, reconfiguration of the shoreline, altered soil-moisture conditions, and associated changes in vegetation patterns and habitat arrangement.

The Khortytsia floodplain responded to the disaster through fast reorganisation of riverine and floodplain habitats. The main developments included separation of some waterbodies from the main channel, ongoing retreat of water, emergence of exposed substrates and sandy forms, and early colonisation of these freshly exposed areas by pioneer vegetation linked to wet habitats (Lisovets et al., 2025). Environmental responses in the higher parts of the floodplain were less immediate than along the former shoreline, but they were still important because reduced moisture availability in the root zone increased pressure on woody vegetation, especially during summer. As these hydrological effects spread beyond the areas of direct water retreat, the floodplain developed a heterogeneous pattern of newly formed and unstable surface states with distinct spectral expression. This complexity is particularly relevant for remote-sensing analysis because it requires approaches that can register land-cover classes not defined in advance. To interpret the 2023 patterns more reliably, they were considered within a broader Sentinel-2 time series spanning 2022–2025, which made it possible to separate ordinary phenological variability from changes associated with the catastrophe. The effects of reservoir drawdown therefore extended beyond the former littoral zone: after the initial loss or isolation of many floodplain waterbodies, further transformation progressed into the inner floodplain through continuing hydrological shifts and successional change (Tutova et al., 2026d).

### Spectral indices

Spectral indices were used as standardised descriptors for characterising vegetation condition and temporal variation, allowing comparisons among observation dates and helping to trace changes in the spectral contribution of vegetation and soil-related surface features (Table 1). Comparisons based on separate indices are commonly applied in studies of vegetation-cover dynamics, and Normalized Difference Vegetation Index (NDVI) is among the most frequently used indicators for this purpose. The equations of the indices included in the analysis, together with their ecological interpretation, are described in the protocol (Kunakh et al., 2025). The following indices were calculated: Aerosol Contrast Index (AC_Index) (Komlyk, Ponomarenko & Zhukov, 2024), Blue-Green Index (BIG2) (Elfanah et al., 2023), Green Leaf Index (GLI) (Louhaichi, Borman & Johnson, 2001), Green NDVI (GNDVI) (Gitelson, Kaufman & Merzlyak, 1996), Land Surface Water Index (LSWI) (Chandrasekar et al., 2010), Modified NDWI (MNDW) (Xu, 2006), Normalized Burn Ratio Index (NBRI) (Seydi et al., 2021), Normalized Difference Bareness Index (NDBaI) (Zhao & Chen, 2005), Normalized Difference Chlorophyll-a (NDChla) (Ha et al., 2017), Normalized Difference Green Chlorophyll Index (NDGCI) (Chaves, Picoli & Sanches, 2020), Normalized Difference Index (NDI) (Wang et al., 2019), Normalized Difference Infrared Index (NDII) (Hardisky, Klemas & Smart, 1983), Normalized Difference Iron Oxide (NDIO) (Yazdi et al., 2013), Blue-normalized difference vegetation index (BNDVI) (Yang et al., 2004), Normalized Difference Red Edge Index (NDRE) (Barnes et al., 2000), Normalized Difference Red-Edge Improved (NDREI) (Barnes et al., 2000), Normalized Difference Tillage Index (NDTI) (Van Deventer et al., 1997), Normalized Difference Total Suspended Matter Index (NDTSM) (Premkumar, Venkatachalapathy & Visweswaran, 2021), Normalized Difference Vegetation Index (NDVI) (Rouse et al., 1974), Normalized Difference Water Index 1 (NDWI1) (McFeeters, 1996), Normalized Difference Water Index 2 (NDWI2) (Gašparović & Singh, 2020), Red–Blue NDVI (RBNDVI) (Wang et al., 2019), Red Edge NDVI 1 (RedEdge_NDVI1) (Xie et al., 2018), Red Edge NDVI 2 (RedEdge_NDVI2) (Xie et al., 2018), Red Edge Difference Index (REDI) (Addabbo et al., 2016), Red Edge Normalized Difference Vegetation Index (RENDVI) (Gitelson, Kaufman & Merzlyak, 1996), Rock Index (RI) (Imbroane, Melenti & Gorgan, 2007), Structure Insensitive Pigment Index (SIPI) (Penuelas, Baret & Filella, 1995), Sentinel-2 Vegetation Salinity Index (SVSI) (Lugassi, Goldshleger & Chudnovsky, 2017), Tasseled cap greenness, brightness, and wetness indexes (TCI GREEN, TCI BRIGHT, TCI WET) (Kauth & Thomas, 1976), Modified soil-adjusted vegetation index (MSAVI) (Huete, 1988; Qi et al., 1994), Chlorophyll green (ClGRN) (Gitelson & Merzlyak, 1997), Visual atmospheric resistance index (VARI) (Gitelson et al., 2002), Modified triangular vegetation index (MTVI2) (Haboudane et al., 2002). The spectral index formulas and their ecological interpretations are presented in Table S1. Spectral indices were calculated from Sentinel-2 MSI Level-2A data for 26 January, 2 March, 26 April, 6 May, 5 June, 20 June, 4 August, 19 August, 8 September, 23 September, 28 September, 3 October, 18 October, 23 October, and 7 November 2023.

**Table 1:** Sentinel spectral indices for ecological analysis.

Index name	Abbreviation	Formula	Ecological meaning
Aerosol contrast index	AC_Index	(B2 − B1)/(B1 + B2)	State of sandy shoreline deposits
Blue-Green index	BIG2	B2/B3	Vegetation–water–soil contrast
Green leaf index	GLI	(2 * B3 − B4 − B2)/2 * B3 + B4 + B2)	Vegetation greenness and cover density
Green NDVI	GNDVI	(B7 − B3)/(B7 + B3)	Chlorophyll-related vegetation vigour
Land surface water index	LSWI	(B5 − B6)/(B5 + B6)	Vegetation and soil moisture
Modified NDWI	MNDW	(B3 − B11)/(B3 + B11)	Open water extent
Normalized burn ratio index	NBRI	(B8 − B12)/(B8 + B12)	Burn-related disturbance and vegetation loss
Normalized difference bareness index	NDBaI	(B6 − B11)/(B6 + B11)	Bare substrate exposure
Normalized difference Chlorophyll-a	NDChla	(B3 − B4)/(B3 + B4)	Chlorophyll-a in water
Normalized difference green Chlorophyll index	NDGCI	(B8a − B3)/(B8a + B3)	Leaf chlorophyll content
Normalized difference Index	NDI	(B12 − B7)/(B12 + B7)	Soil salinity signal
Normalized difference infrared index	NDII	(B8 − B11)/(B8 + B11)	Vegetation water content
Normalized difference iron oxide	NDIO	(B4 − B2)/(B2 + B4)	Iron oxide exposure
Blue-normalized difference vegetation index	BNDVI	(B12 − B1)/(B12 + B1)	Stressed or sparse vegetation greenness
Normalized difference red edge index	NDRE	(B7 − B5)/(B7 + B5)	Chlorophyll status and vegetation iistress
Normalized difference red-edge improved	NDREI	(B8 − B5)/(B8 + B5)	Early chlorophyll change and vegetation stress
Normalized difference tillage index	NDTI	(B11 − B12)/(B11 + B12)	Disturbed or exposed dry soil surface
Normalized difference total suspended matter index	NDTSM	(B7 − B2)/(B7 + B2)	Suspended matter in water
Normalized difference vegetation index	NDVI	(B8 − B4)/(B8 + B4)	Vegetation greenness and productivity
Normalized difference water index 1	NDWI1	(B3 − B8)/(B3 + B8)	Open water and surface wetness
Normalized difference water index 2	NDWI2	(B5 − B12)/(B5 + B12)	Soil and vegetation moisture
Red–Blue NDVI	RBNDVI	(B8 − (B5 + B1))/(B8 + B5 + B1)	Mixed vegetation–soil salinity signal
Red edge NDVI 1	RedEdge_NDVI1	(B6 − B4)/(B6 + B4)	Early vegetation stress and chlorophyll status
Red edge NDVI 2	RedEdge_NDVI2	(B7 − B4)/(B7 + B4)	Dense-canopy chlorophyll status
Red edge difference index	REDI	(B7 − B6)/(B7 + B6)	Subtle chlorophyll variation
Red Edge Normalized Difference Vegetation Index	RENDVI	(B7 − B5)/(B7 + B5)	Vegetation condition in dense cover
Rock Index	RI	(B3 − B12)/(B3 + B12)	Rocky or dry exposed surfaces
Structure insensitive pigment index	SIPI	(B8 − B2)/(B8 − B4)	Pigment stress signal
Sentinel–2 vegetation salinity index	SVSI	(B4 − B2)/(B5 + B11)	Salinity-related vegetation stress
Tasseled cap greenness	(TCI GREEN)	TCI GREEN = −0.2848 · B02 − 0.2435 · B03 − 0.5436 · B04 + 0.7243 · B08 + 0.0840 · B11 − 0.1800 · B12	Vegetation vigour
Tasseled cap brightness	(TCI BRIGHT)	TCI BRIGHT = 0.3037 · B02 + 0.2793 · B03 + 0.4743 · B04 + 0.5585 · B08 + 0.5082 · B11 + 0.1863 · B12	Surface brightness and substrate exposure
Tasseled cap wetness	(TCI WET)	TCI WET = 0.1509 · B02 + 0.1973 · B03 + 0.3279 · B04 + 0.3406 · B08 − 0.7112 · B11 − 0.4572 · B12	Surface and vegetation moisture
Modified soil-adjusted vegetation index	(MSAVI)	$MSAVI = {{2B08 + 1 - \sqrt {{{\left( {2B08\ +\ 1} \right)}^2} - 8\left( {B08 - B04} \right)} } \over 2}$	Vegetation signal with reduced soil background
Chlorophyll green	(ClGRN)	$CIGRN = {{B08} \over {B03}} - 1$	Chlorophyll and photosynthetic activity
Visual atmospheric resistance index	(VARI)	$VARI = {{B03\ -\ B04} \over {B03\ +\ B04\ -\ B02}}$	Visible-range vegetation greenness
Modified triangular vegetation index	(MTVI2)	$MTVI2 = {{1.5*\left( {1.2*\left( {B08\ -\ B03} \right)\ -\ 2.5*\left( {B04\ -\ B03} \right)} \right)} \over {\sqrt {{{\left( {2*B08\ +\ 1} \right)}^2}\ -\ \left( {6*B08\ -\ 5*\sqrt {B04} } \right) - 0.5} }}$	Canopy chlorophyll signal

### Preparation of predictors and computational workflow

The analysis used multiple sets of spectral-index raster data created from Sentinel-2 MSI Level-2A images, with each observation date represented by its own set of single-band GeoTIFF files. Data preparation and modeling were performed in R, primarily using the terra package for raster processing and the MASS package for implementing linear discriminant analysis. Before comparing data across time, all rasters were aligned to a common spatial framework defined by a reference layer. This preparation involved careful steps such as verifying coordinate systems, reprojecting data when needed, clipping data to the standard study area, and aligning all data to a shared grid and resolution. Continuous variables were resampled using bilinear interpolation, while categorical layers, like the Scene Classification Layer (SCL), were resampled using nearest-neighbor methods. Only predictors available for every date in the analysis were kept to ensure a consistent set of variables throughout the time series. To standardize all dates on the same scale, standardization parameters were estimated jointly across all images rather than separately for each date. For each index, the mean and standard deviation were calculated from a combined random sample drawn from the entire temporal series. Each raster layer was then standardized using these common parameters, reducing the impact of differences in variance or numerical range between dates. A single reference sample of randomly selected pixels—usually about 20,000 points—was created, ensuring that each location had valid data for all retained predictors on all dates. The standardized values extracted at these points were used for clustering, while the original raw index values were kept for ecological interpretation of the resulting cluster profiles.

### Spectral clustering and temporal cluster refinement

Clustering was carried out in R using the hclust function for hierarchical agglomerative clustering, with the number of clusters chosen based on quality criteria (R Core Team, 2025). The process involved two main stages. Initially, for the first date, principal component analysis (PCA) was performed, and the first two principal components (PC1 and PC2) were used to conduct the initial hierarchical clustering. Then, the influence of PC1 and PC2 was removed from the full set of predictors through regression, and PCA was applied to the residual data to uncover components that capture less dominant but ecologically significant variations, especially those related to moisture, soil characteristics, or vegetation conditions. The number of residual components retained was decided using the “elbow” method on the eigenvalue plot (Cattell, 1966). The variability within the main clusters was evaluated in the combined space of PCA and residual PCA features using an integrated variability score based on four metrics: the average distance of points to the cluster centroid, the 95th percentile of these distances, the trace of the within-cluster covariance matrix, and its log-determinant. Clusters with variability scores above the average were selected for further subdivision based on their residual PCA scores. This secondary clustering used Euclidean distance, typically with Ward’s D2 linkage, and the optimal number of clusters (k) was determined by the Calinski–Harabasz index (Caliñski & Harabasz, 1974). For later dates, measures of within-cluster heterogeneity were computed in the combined PCA and residual PCA feature space, while maintaining the cluster structure from the previous step. Subclustering was then selectively applied only to the most heterogeneous clusters and only when certain predefined conditions were met, to avoid excessive fragmentation and overly complex solutions. After iterative refinement, a correspondence table linking clusters across dates was created, and cluster labels were updated so that main branches kept the codes of their parent clusters, while new branches received new codes. At this point, the analysis produced temporally connected spectral clusters defined in feature space, before their spatial mapping and ecological interpretation.

### Extrapolation of cluster labels to full rasters

The process of assigning cluster labels back onto the raster images was carried out by training a classifier using the reference points and then applying it to every pixel. This approach allowed for the creation of complete cluster maps for each date. Linear discriminant analysis was used within the same feature space applied for heterogeneity evaluation, which included PC1–PC2 and residual principal component scores. The associated dataset (Tutova et al., 2026a) contains the complete set of land-surface classification results for all analyzed Sentinel-2 dates, including the spatial cluster maps that served as the foundation for temporal comparison and habitat interpretation in this research. To evaluate the reliability of extending cluster labels from the common sample to the entire rasters, an internal validation procedure was conducted. The common-sample points were split into training and testing groups, ensuring that the test points were not used during model training. The linear discriminant classifier, used for predictions across the raster, was first trained on the training subset and then tested on the independent test subset. Predictive performance was measured using confusion matrices and summary statistics such as overall accuracy, balanced accuracy, and macro-F1 score. This process was considered an internal validation of transferring labels from the common sample to the raster data, rather than an external ground-truth validation for every raster cell. Ultimately, this step produced full-coverage spatial maps of the cluster structure for each date analyzed.

### Ecological interpretation of mapped clusters using EUNIS

Habitat types were identified and spectral clusters named by combining index-based spectral data, spatial context, and ecological site features, followed by matching with the EUNIS habitat classification. Since direct field surveys were not possible during the critical phase of the 2023 disaster, fieldwork was carried out in 2024 and 2025, resulting in 878 vegetation records. Based on these records, a preliminary inventory of the vegetation cover in the southern part of Khortytsia Island was created, which is included in the related dataset (Lisovets et al., 2026). Therefore, habitat identification relied on information about vegetation associations, geomorphology, and physiognomy. These data were not used as immediate validation of the 2023 satellite classifications but rather to aid the ecological interpretation of the mapped clusters and their successional changes. Although post-disaster habitats are highly dynamic, the temporal gap was addressed by using ecological indicators that remain meaningful over time, such as the age structure of vegetation—particularly woody plants—the balance of annual *vs*. perennial species, and other signs of succession. Given that the disaster generated new, highly dynamic, and structurally unstable surface features, cluster interpretation was performed through expert analytical cross-referencing between spectral clusters and habitat types, instead of direct automatic classification.

For each cluster, a brief profile was created. This included the distribution of index values at key reference points, represented by medians and quantiles. The cluster’s location within both the principal component and residual principal component spaces was also recorded, capturing major and minor sources of variation. Additionally, the spatial pattern on the map was considered, including its relationship to features such as the shoreline, floodplain waterbodies, newly exposed shallow areas, or elevated parts of the floodplain. The cluster’s temporal behavior was also incorporated, noting aspects like stability and changes over time. It was proposed that, based on these characteristics, clusters could be interpreted as functional surface types identifiable directly from spectral data and landscape position. The surface types examined included open water; littoral and aquatic-related vegetation; bare or sparsely vegetated ground; herbaceous cover of varying densities; shrublands; woody vegetation; and transitional mosaics. The EUNIS classification system was then applied to the study area using a “general-to-specific” method. Initially, the broader EUNIS category was assigned based on dominant spectral features and landscape context. When sufficient information was available, more specific classes were determined using key indicators such as wetness and hydrological conditions, substrate exposure and dryness, overall greenness and productivity, vegetation health, and seasonal patterns of change. When a cluster represented transitional or mixed states typical of post-disaster succession, a cautious approach was taken by assigning it to a broader EUNIS category or labeling it as a complex or transitional type, thus avoiding overly detailed classifications. The established “cluster → EUNIS habitat” connections were then carefully examined to verify their spatial consistency (*i.e*., whether the cluster locations matched the expected ecotopes), seasonal consistency (*i.e*., whether their changes aligned with seasonal variations in water availability and vegetation growth), and ecological plausibility regarding successional changes. The cluster names were designed to be both informative in terms of spectral data and meaningful ecologically. Each class received a stable identifier that remained consistent throughout its temporal development, along with a brief description of the surface type and, when relevant, a reference to the corresponding EUNIS level, provided either as a code or habitat name. This reference could be included directly in the label or listed separately in a correspondence table. In the next phase of gated subclustering, the resulting subclusters retained the base name of their parent class, with an added qualifier indicating differences in factors such as wetness, substrate exposure, or vegetation density.

### Assessing the discriminatory power of spectral indices

The informational value of the spectral indices was evaluated from two complementary angles. For each cluster, the top indices were determined as those exhibiting the greatest differences in average standardized values between that cluster and all other pixels, measured as effect sizes in z-scores. This resulted in a clear list of indices that most effectively differentiated a specific surface or habitat type on a given date. The overall importance of the indices was measured by combining their contributions to the simultaneous separation of all clusters. The indices were ranked based on how strongly they were associated with the discriminant axes of the classification (which included the main PCA components and the residual-based secondary components), and their contributions were normalized into dimensionless weights that summed to one. The linked dataset (Tutova et al., 2026b) provides the date-specific rankings and weights of the spectral indices according to their discriminatory power for cluster differentiation, thereby documenting the quantitative basis for the interpretation of index importance reported in this study.

## Results

### Dynamics of the number of primary and secondary clusters across the periods of the year

The number of primary clusters remained constant at three throughout the year (Table 2). No secondary clusters were detected during periods 1 and 2, indicating limited discriminative capacity for further differentiation of surface types. From period 3 onward, secondary clusters emerged and progressively increased, reaching 15 in periods 10 to 15. This trend reflects growing within-cluster heterogeneity among the primary clusters. Consequently, the total number of clusters increased, while the number of unsplit primary clusters decreased to zero from period 7 onward, when all primary clusters exhibited secondary sub-clustering.

**Table 2:** Dynamics of the number of primary and secondary clusters across the periods of the year.

Period	Number of primary clusters	Number of secondary clusters	Total number of clusters	Number of unsplit primary clusters
1	3	0	3	3
2	3	0	3	3
3	3	4	7	1
4	3	8	11	1
5	3	8	11	1
6	3	8	11	1
7	3	11	14	0
8	3	13	16	0
9	3	13	16	0
10	3	15	18	0
11	3	15	18	0
12	3	15	18	0
13	3	15	18	0
14	3	15	18	0
15	3	15	18	0

### Classification accuracy

The reliability of extrapolating cluster labels from the common sample to full-raster prediction was assessed through internal holdout validation, employing the same classification rule used for raster-wide mapping. This validation framework aligns with the methodological procedure applied to evaluate the transfer from the common sample to the full raster, incorporating overall accuracy, balanced accuracy, and macro-F1 as summary metrics. For the 2023 time series, classification accuracy remained generally high across the 15 analysed dates. The mean overall accuracy was 0.870 ± 0.047, mean balanced accuracy 0.832 ± 0.046, and mean macro-F1 0.802 ± 0.046. Across individual dates, overall accuracy ranged from 0.745 to 0.945, balanced accuracy from 0.699 to 0.879, and macro-F1 from 0.680 to 0.863. These results indicate that the transfer of class labels from the common sample to the full raster was generally robust, although classification performance varied between dates and was lower for some temporally more complex scenes. Among the dates most relevant to the acute post-catastrophic phase represented in the validation output, performance remained within an acceptable to high range. On 5 June 2023, overall accuracy was 0.917, balanced accuracy 0.782, and macro-F1 0.729; on 20 June 2023, the corresponding values were 0.852, 0.811, and 0.799. For the later summer dates included in this validation series, accuracy reached 0.866, 0.842, and 0.820 on 4 August 2023, and 0.903, 0.874, and 0.863 on 19 August 2023. Taken together, these values support the use of the resulting raster maps for interpreting habitat dynamics in 2023, while also indicating that some dates require more cautious interpretation than others.

### Genealogy of Earth’s surface types

From the beginning of the season, differentiation of Earth’s surface types occurs, which can be categorised in terms of habitat types (Fig. 1). Initially, one may conditionally consider a state of minimal distinction among surface types, when it is reasonable to delineate only land, open water, and the shoreline zone (Fig. S1). During period 2, the shoreline differentiated into sandy beach and littoral zone, while the land differentiated into floodplain forests, floodplain shrublands, and floodplain meadows. In period 3, the number of distinct surface types increased to 12, rising to 14 in period 4. A further increase occurred only in period 7, reaching 16 types, and the final increase took place in period 9, reaching 18 types, after which the number of types remained unchanged until the end of the growing season.

**Figure 1:**
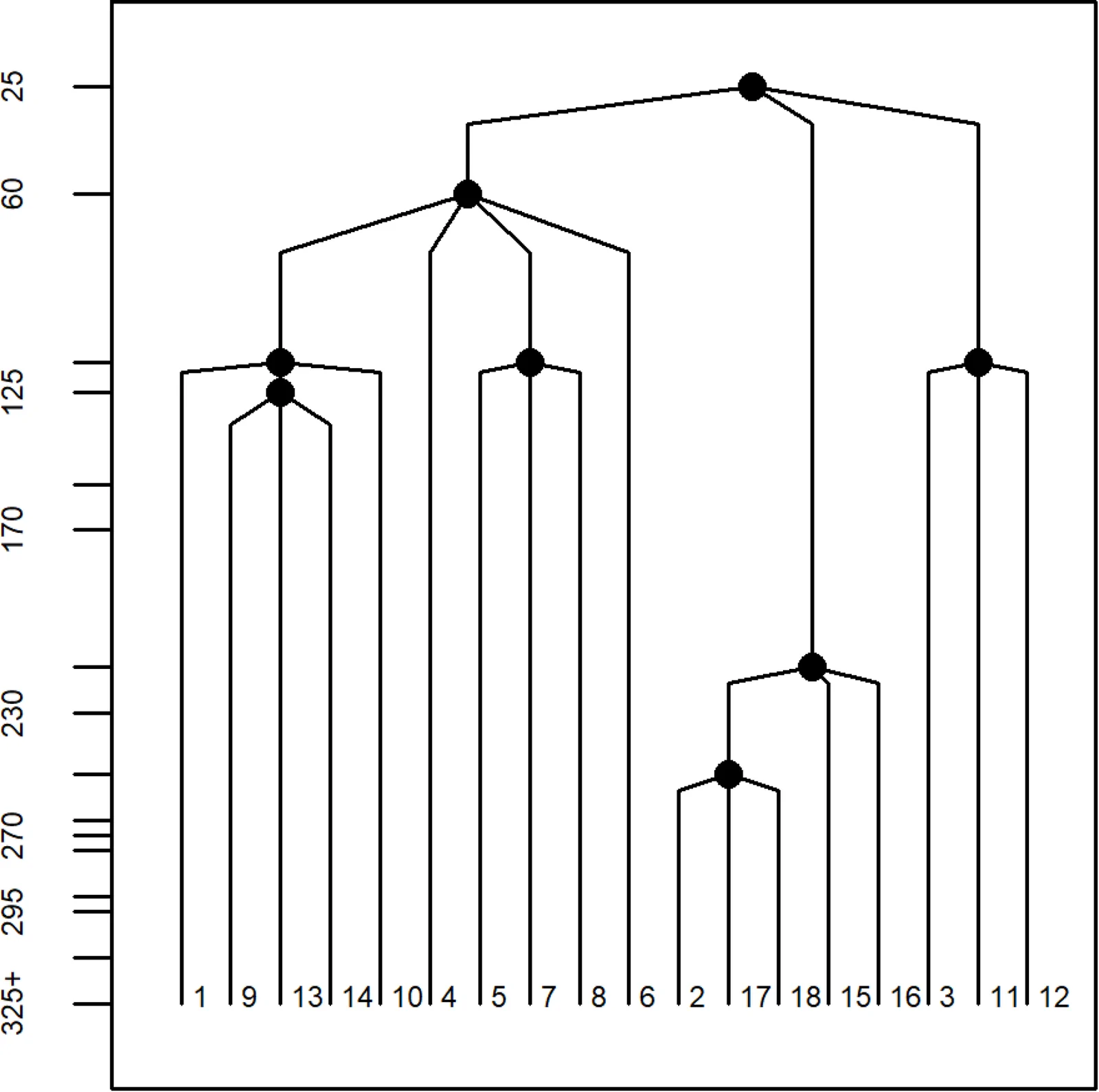
Dynamics of differentiation of Earth’s surface types in terms of habitat types. The y-axis shows the number of days since the beginning of the year (day of year), the x-axis: 1—floodplain forests (T11 + T13); 2—C2.3 Permanent non-tidal, smooth-flowing watercourses; 3—littoral zone (Q51 and C3.6); 4—floodplain shrublands (F9.35 or F9.12); 5—floodplain grasslands/meadows (R35 Moist or wet mesotrophic to eutrophic hay meadow and R37 Meadows on nutrient-poor soils (both nitrogen and phosphorus), wet much of the year, may dry superficially in summer); 6—C3.61 Unvegetated river sand banks; 7—C3.6 Unvegetated or sparsely vegetated shores with soft or mobile sediments; 8—R11 Pannonian & Pontic sandy steppe; 9—Q61 Periodically exposed shore with stable, eutrophic sediments with pioneer or ephemeral vegetation; 10—S912 Lowland and collinar riverine *Salix* scrub; 11—Q511 *Phragmites australis* beds; 12—Q51D2 *Typha angustifolia* beds normally without free-standing water; 13—E5.43 Shady woodland edge fringes; 14—C3.53 Euro-Siberian annual river mud communities; 15—C3.65 Exposed unvegetated freshwater lake muds; 16—C3.5 Periodically inundated shores with pioneer and ephemeral vegetation.

### Importance of indices for differentiating habitat types

The spectral indices most informative for discriminating habitat types follow the sequence below in decreasing order of importance: NDNIRBlue (0.0423) ← MNDW (0.0421) ← GNDVI (0.0418) ← NDWI1 (0.0418) ← NDTSM (0.0414) ← NDGCI (0.0414) ← CIGRN (0.0394) ← RI (0.0390) ← NDVI (0.0389) ← NDTI (0.0381). Throughout the year, the indices prioritised for differentiating habitat types change seasonally. In late winter and early spring, indices sensitive to water and wetness dominate (MNDW, NDWI2, TCI_WET). In late spring, indices sensitive to overall greenness increase in importance, particularly NDVI. At the beginning of summer, indices sensitive to biomass, canopy density, and vegetation productivity prevail (MSAVI, MTVI2, GNDVI, CIGRN), with a notable contribution from the red-edge region of the spectrum. In mid and late summer, indices sensitive to chlorophyll content and vegetation condition become more prominent (NDRE, RENDVI), as do those related to surface brightness and dryness (TCI_BRIGHT, *etc*.). In autumn, the contribution of indices sensitive to exposed substrate and cover degradation increases (NDTI, NDBaI, NBRI). Simultaneously, for floodplain and riparian habitats, wetness- and water-related indices again become important. The global weights of the highest-ranking indices indicate that habitat differentiation in this landscape system relied on vegetation-related indices together with indices sensitive to water conditions, moisture, and substrate properties.

### Habitat structure changes resulting from the disaster

The destruction of the Kakhovka Hydroelectric Power Plant dam and the consequent abrupt drop in water levels in the Kakhovka Reservoir caused catastrophic changes to the living conditions of floodplain biota. The identified habitat types were further supported by the composition of vegetation associations recorded in the field, which provided independent ecological confirmation of the habitat classification and its interpretation (Table 3). Remote sensing enables the monitoring of changes associated with the transformation of both the Dnipro River channel ecosystems and the floodplain ecosystems. In floodplain systems, the decline in water levels proved fatal: within just a few days, the water level fell sharply, and floodplain waterbodies that had been connected to the river channel were transformed into lake-like basins with significantly reduced surface area and water volume (Fig. 2). The western shore of the island is steeper; therefore, changes there were considerably less pronounced than in the eastern part of the island. In the east, extensive shallow-water areas became a flattened section of the shoreline. The process of water-level decline and progressive shallowing of the waterbodies continued throughout almost the entire growing season. Additionally, in late summer and early autumn, shoreline transformation processes also became noticeable on the western side of the island. The most pronounced early changes (Table 4) included a sharp increase in unvegetated river sand banks from 1.4% to 4.9%, periodically exposed eutrophic shores with pioneer or ephemeral vegetation from 6.0% to 8.4%, and lowland riverine *Salix* scrub from 1.8% to 3.5%, accompanied by a marked decline in floodplain forests from 20.3% to 15.5% and *Phragmites australis* beds from 5.2% to 2.5%.

**Table 3:** Distribution of vegetation associations across the identified habitat categories, %.

Association**	Habitat*														
	1	3	4	5	6	7	8	9	10	11	12	13	14	15	16
1	–	–	–	–	–	81.8	–	–	–	–	–	–	3.8	–	20.6
2	–	38.6	–	–	–	18.2	–	–	–	–	–	–	7.7	10.0	36.5
3	–	–	–	–	46.7	–	–	–	–	–	–	–	–	15.0	25.4
4	–	–	–	–	30.0	–	–	2.2	–	–	–	–	7.7	35.0	17.5
5	–	–	–	–	10.0	–	–	20.0	–	–	65.2	–	–	10.0	–
6	–	–	–	37.5	6.7	–	–	26.7	–	–	21.7	–	–	5.0	–
7	–	6.8	–	29.2	3.3	–	–	28.9	14.3	–	13.0	2.0	34.6	–	–
8	–	6.8	22.2	25.0	3.3	–	–	15.6	30.6	–	–	6.0	46.2	5.0	–
9	12.6	6.8	26.7	8.3	–	–	–	6.7	55.1	11.8	–	30.0	–	10.0	–
10	6.3	6.8	31.1	–	–	–	–	–	–	14.7	–	26.0	–	–	–
11	5.9	–	13.3	–	–	–	–	–	–	29.4	–	–	–	–	–
12	11.2	–	6.7	–	–	–	–	–	–	26.5	–	–	–	5.0	–
13	12.6	–	–	–	–	–	–	–	–	8.8	–	–	–	5.0	–
14	13.8	–	–	–	–	–	–	–	–	5.9	–	–	–	–	–
15	5.9	–	–	–	–	–	–	–	–	–	–	20.0	–	–	–
16	16.7	–	–	–	–	–	–	–	–	–	–	16.0	–	–	–
17	14.9	13.6	–	–	–	–	–	–	–	–	–	–	–	–	–
18	–	11.4	–	–	–	–	18.5	–	–	–	–	–	–	–	–
19	–	9.1	–	–	–	–	37.0	–	–	2.9	–	–	–	–	–
20	–	–	–	–	–	–	44.4	–	–	–	–	–	–	–	–

**Note:**

Habitat*: 1—floodplain forests (T11 + T13); 2—C2.3 Permanent non-tidal, smooth-flowing watercourses; 3—littoral zone (Q51 and C3.6); 4—floodplain shrublands (F9.35 or F9.12); 5—floodplain grasslands/meadows (R35 Moist or wet mesotrophic to eutrophic hay meadow and R37 Meadows on nutrient-poor soils (both nitrogen and phosphorus), wet much of the year, may dry superficially in summer); 6—C3.61 Unvegetated river sand banks; 7—C3.6 Unvegetated or sparsely vegetated shores with soft or mobile sediments; 8—R11 Pannonian & Pontic sandy steppe; 9—Q61 Periodically exposed shore with stable, eutrophic sediments with pioneer or ephemeral vegetation; 10—S912 Lowland and collinar riverine *Salix* scrub; 11—Q511 *Phragmites australis* beds; 12—Q51D2 *Typha angustifolia* beds normally without free-standing water; 13—E5.43 Shady woodland edge fringes; 14—C3.53 Euro-Siberian annual river mud communities; 15—C3.65 Exposed unvegetated freshwater lake muds;16—C3.5 Periodically inundated shores with pioneer and ephemeral vegetation. Association**–Ruderal vegetation: 1—*Bromo tectorum-Corispermetum leptopteri* Sissingh et Westhoff ex Sissingh 1950 corr. Dengler 2000, 2—*Chenopodietum stricti* (Oberd. 1957) Passarge 1964, 3—*Portulacetum oleracei* Felföldy 1942, 4—*Amarantho retroflexi-Echinochloetum cruris-galli* Bagrikova 2005; Wetland: 5—*Typhetum angustifoliae* Pignatti 1953, 6—*Rumici maritimi-Ranunculetum scelerati* Oberd. 1957, 7—BC *Cyperus fuscus* [*Eleocharition soloniensis*], 8—DC *Oxybasis rubra* [*Phragmitetalia*]; Shrublands: 9—*Populetum nigro-albae Slavnić* 1952, 10—DC *Amorpha fruticosa* [*Phragmition australis*], Pioner vegetation: 11—DC *Cirsiumarvense* [*Phragmition australis*]; 12—DC *Erigeroncanadensis* [*Phragmition australis*], 13—*Erigeronto*-*Lactucetumserriolae* Lohmeyer in Oberd. 1957; Forest and forest edges: 14—*Salicetum albae*Issler 1926, 15—*Symphyto officinalis-Anthriscetum sylvestris* Passarge 1975, 16—*Myosotido spars і florae-Alliarietum petiolatae* Gutte 1973, 17—DC *Stellaria media* [*Aceri tatarici-Quercion*]; Psammophytic: 18—DC *Viciavillosa* [*Artemisioarenariae-Festucionbeckeri*], 19—*Secalisylvestri-Brometumtectorum* Hargitai 1940, 20—*Secaletum sylvestre* Popescu et Sanda 1973.

**Figure 2:**
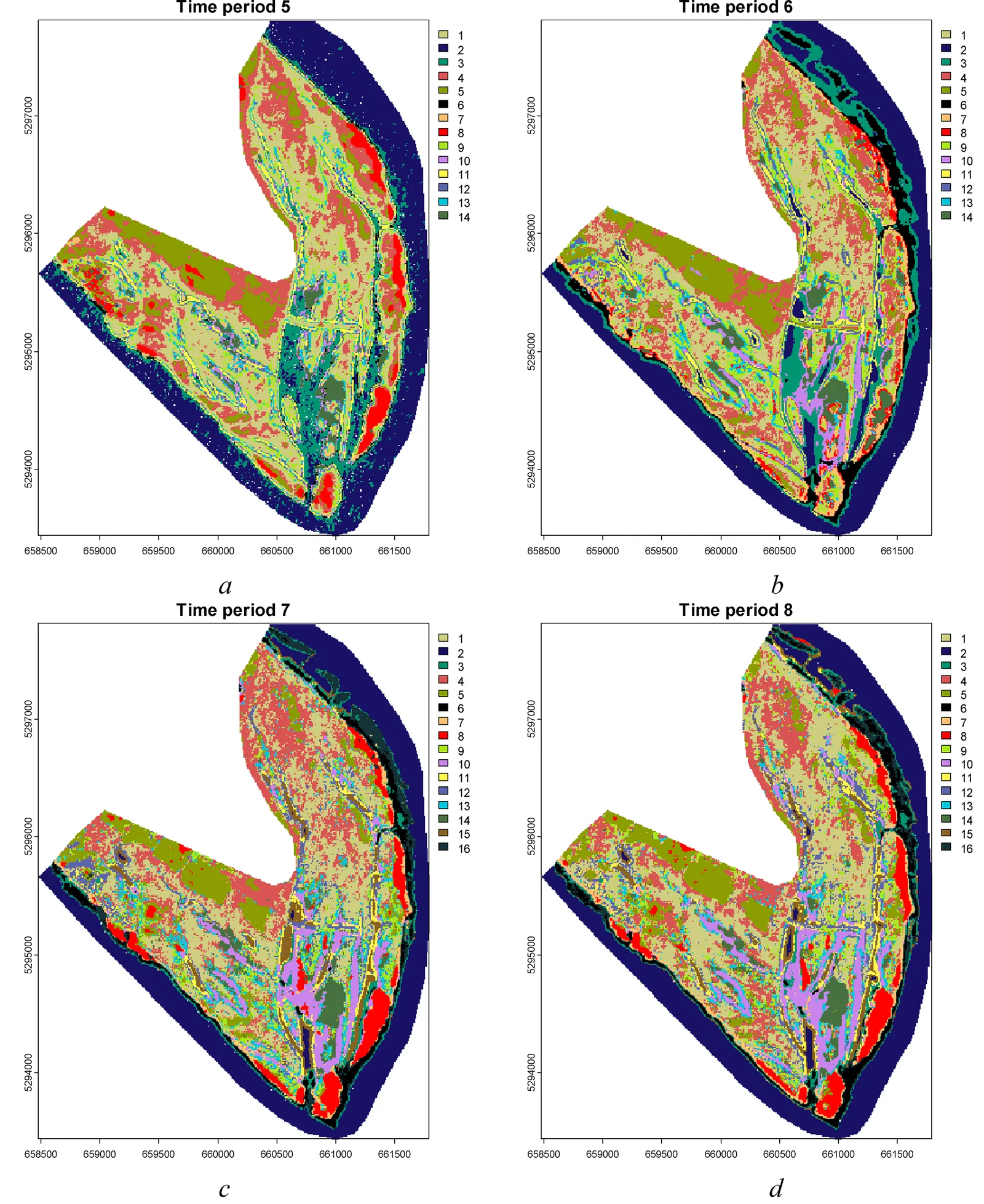
Spatial distribution of land-surface types. (A) 5 June (the last date before the catastrophe), (B) 20 June (first image after the disaster); (C) 4 August; (D) 19 August: 1—floodplain forests (T11 + T13); 2—C2.3 Permanent non-tidal, smooth-flowing watercourses; 3—littoral zone (Q51 and C3.6); 4—floodplain shrublands (F9.35 or F9.12); 5—floodplain grasslands/meadows (R35 Moist or wet mesotrophic to eutrophic hay meadow and R37 Meadows on nutrient-poor soils (both nitrogen and phosphorus), wet much of the year, may dry superficially in summer); 6—C3.61 Unvegetated river sand banks; 7—C3.6 Unvegetated or sparsely vegetated shores with soft or mobile sediments; 8—R11 Pannonian & Pontic sandy steppe; 9—Q61 Periodically exposed shore with stable, eutrophic sediments with pioneer or ephemeral vegetation; 10—S912 Lowland and collinar riverine *Salix* scrub; 11—Q511 *Phragmites australis* beds; 12—Q51D2 *Typha angustifolia* beds normally without free-standing water; 13—E5.43 Shady woodland edge fringes; 14—C3.53 Euro-Siberian annual river mud communities; 15—C3.65 Exposed unvegetated freshwater lake muds; 16—C3.5 Periodically inundated shores with pioneer and ephemeral vegetation.

**Table 4:** Area share of classified habitat categories on Khortytsia Island before and after the catastrophe in June–August 2023.

No.	Category	5 June 2023 (before catastrophe)	20 June 2023 (first image after disaster)	4 August 2023	19 August 2023
1	Floodplain forests (T11 + T13)	20.3	15.5	15.8	17.1
2	C2.3 Permanent non-tidal, smooth-flowing watercourses	18.5	19.6	18.4	18.9
3	Littoral zone (Q51 and C3.6)	8.2	7.8	2.8	2.8
4	Floodplain shrublands (F9.35 or F9.12)	14.9	13.8	13.2	12.1
5	Floodplain grasslands/meadows (R35, R37)	8.7	8.0	7.1	6.8
6	C3.61 Unvegetated river sand banks	1.4	4.9	4.0	4.2
7	C3.6 Unvegetated or sparsely vegetated shores with soft or mobile sediments	2.4	3.8	3.0	3.2
8	R11 Pannonian & Pontic sandy steppe	3.5	2.6	4.3	4.4
9	Q61 Periodically exposed shore with stable, eutrophic sediments with pioneer or ephemeral vegetation	6.0	8.4	5.6	5.9
10	S912 Lowland and collinar riverine Salix scrub	1.8	3.5	5.5	5.8
11	Q511 *Phragmites australis* beds	5.2	2.5	2.5	2.6
12	Q51D2 *Typha angustifolia* beds normally without free-standing water	2.6	3.3	3.8	4.4
13	E5.43 Shady woodland edge fringes	3.9	3.0	5.5	4.2
14	C3.53 Euro-Siberian annual river mud communities	2.7	3.3	2.9	2.6
15	C3.65 Exposed unvegetated freshwater lake muds	–	–	2.7	2.9
16	C3.5 Periodically inundated shores with pioneer and ephemeral vegetation	–	–	2.8	2.4

## Discussion

### Advantages of unsupervised classification for disaster monitoring

Catastrophic changes occur against the backdrop of regular seasonal variations in the composition of the landscape cover. Although the most immediate changes were concentrated along the former land–water interface and in floodplain waterbodies, the consequences ultimately affected the entire floodplain territory of the island, with later-stage succession proceeding through willow–poplar expansion, local development of Amorpha thickets, and subsequent pyrogenic transformation in later years (Lisovets et al., 2025; Tutova et al., 2025). The spectral “signature” of floodplain ecosystems, as detected by remote sensing, arises from the interaction between vegetation, soil cover, and waterbodies. Their seasonal dynamics create a complex mosaic of cover types that vary in both spatial configuration and spectral characteristics. In winter, when vegetation is physiologically dormant, landscape cover differentiation is minimal, essentially limited to distinguishing open water, dry land, and the shoreline zone. A more detailed differentiation is possible, but it depends on the trade-off between thematic resolution and the reliability of selecting a specific clustering solution (Zelenova et al., 2025). The Calinski–Harabasz (CH) index serves as an informative criterion for selecting the optimal number of clusters because it directly contrasts between-cluster separation with within-cluster compactness, aligning with intuitive expectations regarding the nature of landscape cover types (Caliñski & Harabasz, 1974). The CH index increases when clusters become simultaneously more compact internally and better separated from one another, with its maximum often representing a reasonable compromise between over-clustering and under-clustering (Li et al., 2016). A practical advantage of the CH index is that it is computationally efficient, scales well to large samples, and naturally complements algorithms such as k-means (Arbelaitz et al., 2013; Nykytiuk & Kravchenko, 2025) or Euclidean-distance hierarchical clustering using Ward’s method (Ikotun, Habyarimana & Ezugwu, 2025). More generally, it is suitable for situations where clusters are expected to be relatively compact in the chosen feature space—an important property for repeated analyses across multiple temporal snapshots of satellite data (Murtagh & Legendre, 2014). However, the CH index has some interpretational caveats: it tends to favour more spheroidal clusters with roughly similar dispersion and may overestimate the number of clusters (k) in the presence of strong density heterogeneity, outliers, or complex (non-spherical) cluster shapes (Moulavi et al., 2014). Therefore, it is advisable to monitor solution stability and the ecological interpretability of the results. Potential alternatives include the silhouette coefficient (Rousseeuw, 1987), the Davies–Bouldin index (Davies & Bouldin, 1979), the gap statistic (Tibshirani, Walther & Hastie, 2001), the Dunn index (Dunn, 1974), information criteria (AIC/BIC) for probabilistic models (*e.g*., Gaussian mixture models) (Akaike, 1974), or stability-based approaches such as resampling or bootstrapping (Ben-Hur, Elisseeff & Guyon, 2001). However, these methods are often substantially more computationally demanding or more sensitive to sample size and distance structure in high-dimensional settings. Given the need for a reproducible, fast, and cross-date comparable rule, the CH index provides a well-justified baseline criterion that yields an objective estimate of the optimal number of clusters without costly procedures, while alternative methods can be employed for validation in ambiguous cases. The most meaningful heuristic criterion for clustering quality is the interpretability of the resulting clusters within the context of the subject domain. Internal statistical optimality indices primarily reflect geometric properties of the partition—such as compactness and separation in the feature space—but alone, they do not guarantee that the identified groups correspond to actual object types. Given the need for a reproducible, fast, and cross-date comparable rule, the CH index provides a well-justified baseline criterion that yields an objective estimate of the optimal number of clusters without costly procedures, while alternative methods can be employed for validation in ambiguous cases (Arbelaitz et al., 2013). A formal sensitivity analysis comparing the Calinski–Harabasz index with alternative validity measures was not included in the present study and should be regarded as an important direction for further methodological refinement. The most meaningful heuristic criterion for clustering quality is the interpretability of the resulting clusters within the context of the subject domain. Therefore, the final selection of a clustering solution should be based on the potential for substantive interpretation and its consistency with independent knowledge. In our case, this criterion involves the ability to reliably associate clusters with habitat types and to reproduce their ecological logic across space and time.

### Stepwise principal component analysis to detect signals masked by vegetation

The ability to differentiate land-cover types improves throughout the growing season as these cover types gradually develop more distinct characteristics. However, increasing plant phytomass reduces the capacity to distinguish soil and water conditions, which significantly contribute to the uniqueness of a given habitat type. Consequently, the signal from non-vegetated components of the Earth’s surface becomes weaker and more diffuse, often being filtered out by statistical procedures as secondary noise. Principal component analysis (PCA) of spectral data is commonly employed to filter data and remove noise (Green et al., 1988). To denoise multispectral imagery, sequential principal component analysis has been proposed (de Carvalho, de Carvalho & Meneses, 2000). In our study, sequential PCA was applied to achieve a more detailed differentiation of land cover through unsupervised classification. Notably, the increasing contribution of secondary principal components over the growing season indicates that edaphic factors become more important for differentiating vegetation cover as the season progresses. Our results demonstrate that combining primary and secondary PCA components provides substantially greater discriminatory power for spectral data. Therefore, relying solely on vegetation indices sensitive to vegetation cover density is insufficient for the accurate classification of vegetation cover types.

### Catastrophic ecosystem changes against the background of phenological dynamics

The greatest differentiation of terrestrial land-cover types occurs in late spring or early summer; however, the disaster caused by the destruction of the Kakhovka HPP dam and the abrupt drop in water levels in the Kakhovka Reservoir triggered a cascade of ecosystem changes. The rapid decline in water levels led to the effective collapse of the floodplain pond system on Khortytsia Island (Fig. 3). This pattern may be indicative of broader processes within the former reservoir area, although the present analysis directly documents only the Khortytsia case study. Our results align with other studies reporting significant climatic and hydrological changes throughout the Kakhovka Reservoir (Pichura et al., 2024; Pichura, Potravka & Boiko, 2025). It is probable that a new floodplain pond system will develop under the altered water level, but the establishment of stable and diverse plant communities within it will require considerable time. These changes are consistent with severe degradation of floodplain-pond habitats reported in external studies, although this specific conclusion is not derived solely from the present remote-sensing analysis. The remaining floodplain ponds now function as temporary waterbodies that dry out. During summer, these ponds overheat, and organic-rich bottom sediments decompose intensively, creating conditions unfavorable for the survival of higher plants and animals.

**Figure 3:**
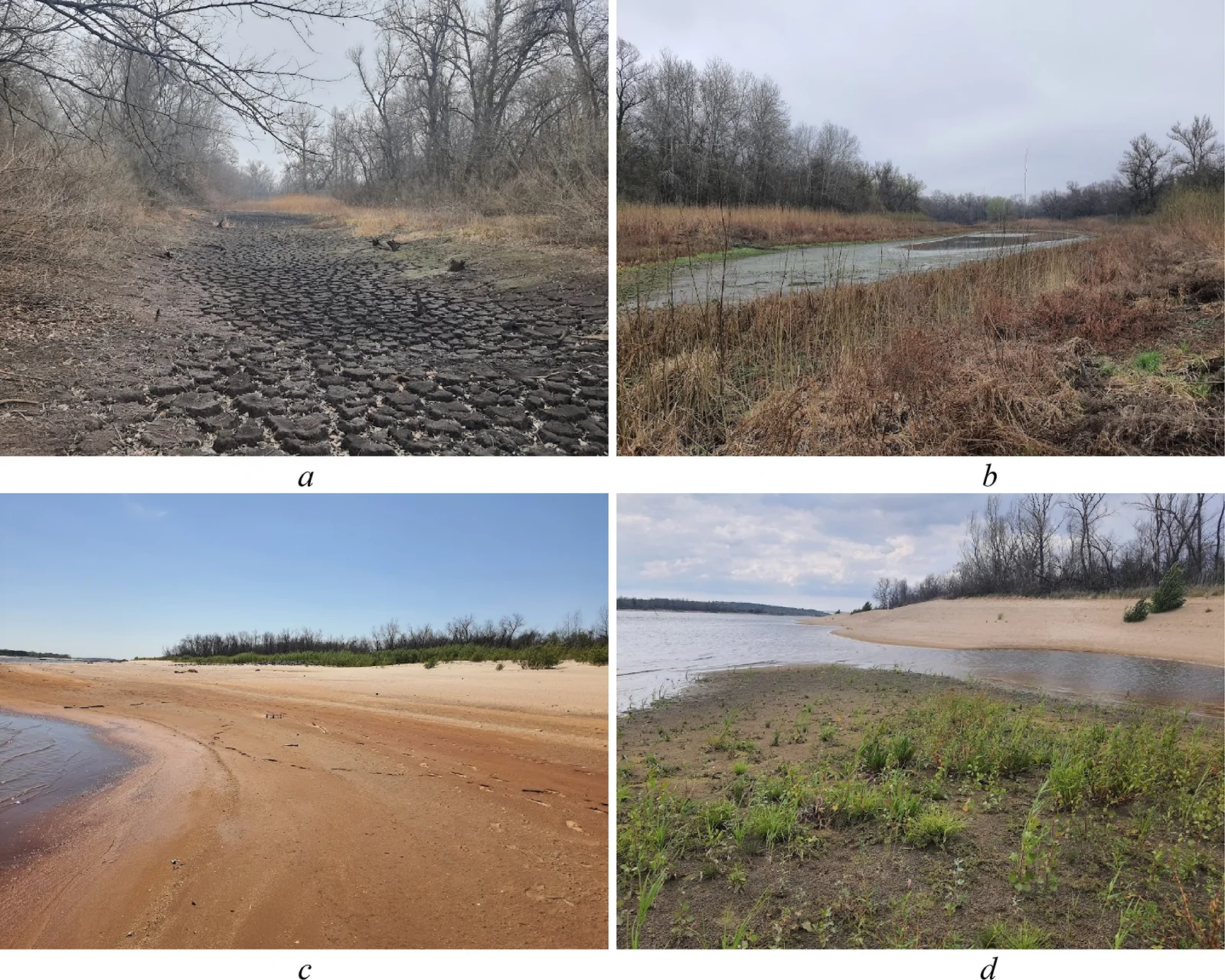
Images of the main types of habitats that underwent transformation after the destruction of the Kakhovka Dam and the abrupt drop in water level in the Kakhovka Reservoir (March 2025). (A) the bottom of a floodplain waterbody that disappeared and has remained unvegetated for a prolonged period; (B) a remnant of a floodplain waterbody with a substantially lowered water level, showing overgrowth of the shores by herbaceous vegetation and willow and poplar thickets; (C) the exposed bed of the Dnipro River channel after water-level recession (eastern part of the island); (D) the exposed bed of the Dnipro River channel after water-level recession (western part of the island).

The dried bottoms of floodplain waterbodies created favorable conditions for the spread of terrestrial vegetation. Intensive growth of willow and poplar communities has been observed in these habitats (Kuzemko et al., 2025), which in turn promotes substantial carbon dioxide sequestration, yielding a pronounced positive ecosystem effect (Tutova et al., 2025). Along the outer margin of the island, where it borders the main channel of the Dnipro River, extensive sandy shores were exposed due to the decline in water levels. The eastern shore of the island is gentler; consequently, shallow waters transformed into large sandy beach areas enriched with organic residues. These locations were rapidly colonized by helophytic and other aquatic-associated macrophytes, as reflected in strong dynamism observed through remote sensing. The western shore is steeper and better drained, creating more extreme conditions for plant establishment. Vegetation encroachment there is relatively slow, and aeolian factors play a substantial role. Importantly, habitats forming in the aftermath of the disaster are characterized by high dynamism and structural uncertainty. Therefore, supervised classification procedures for remote-sensing imagery are of limited applicability under such conditions. In such situations, unsupervised classification approaches become particularly useful, as they allow the identification of unexpected spectral objects that indicate new habitat types emerging through post-catastrophe succession.

It should be noted that the disaster triggered a cascade of transformations that will continue for many years. In the time series of images from the first year, ongoing processes of continuous habitat-structure changes within floodplain waterbodies and along the banks of the Dnipro River are clearly evident. Slower, yet equally significant, transformations are occurring on the relatively elevated parts of the floodplain margins. Woody communities were also affected; however, their inherent conservatism somewhat slows the pace of change. Undoubtedly, the decline in water levels substantially altered the typical moisture dynamics of the root-zone soil layer, which is particularly important for the vegetation of woody plants. The low capillary properties of the sandy alluvial soils of the floodplain make a stable seasonal water-level regime during the critical summer months essential for sustaining floodplain forests. A sharp shift in this regime markedly worsens tree growing conditions, increasing the risk of deterioration in the physiological status of stands or the emergence of other catastrophic phenomena such as fires or pest outbreaks. Indeed, floodplain fires and pest population outbreaks occurred in the years following the disaster.

### Ecological consequences of post-catastrophic habitat transformation

The ecological consequences of the catastrophe can be interpreted in two complementary perspectives. On the one hand, the drawdown caused an abrupt collapse of the floodplain system that had persisted for approximately 70 years under reservoir conditions. The destruction and hydrological disconnection of floodplain waterbodies led to the loss of aquatic habitats and their associated biota, while the exposure of organic-rich bottom sediments initiated their rapid oxidation and burning potential. On the other hand, these changes may also be viewed as the beginning of a longer-term reorganisation towards ecosystem states more similar to those that existed before reservoir formation. The opening and fragmentation of floodplain forests, although destructive in the short term, increased landscape heterogeneity and may create new ecological niches during subsequent successional stages. Thus, the identified habitat types reflect not only catastrophic habitat loss, but also the emergence of new ecological trajectories with potentially important consequences for birds, amphibians, mammals, and other components of the island biota. Observations from spring 2026 indicate signs of renewed flooding dynamics in some waterbodies, which may be interpreted as an early marker of recovery mechanisms in the floodplain system under the lowered erosion base. This suggests that at least part of the former reservoir territory retains the capacity for progressive hydrological and ecological reorganisation. Over time, such processes may contribute to the emergence of a new dynamic equilibrium of the ecosystem. In this context, the territory of the former Kakhovka Reservoir may be regarded as a promising area for biodiversity conservation and, potentially, for the establishment of a national park.

### Limitations and research perspectives

Several limitations of the present study should be acknowledged. Although the analysis encompasses both the pre-disaster period and the acute post-disaster phase within 2023, it does not employ a comprehensive phenology-based baseline framework involving multi-year comparisons with quasi-stationary pre-event conditions. Such an approach would be highly desirable for a more rigorous distinction between catastrophe-driven transformations and ordinary seasonal dynamics. However, within the context of unsupervised classification, this task entails addressing a substantially more complex methodological challenge, namely the robust matching of cluster structures across different years without predefined training labels. Since post-disaster habitats are highly dynamic and cluster identities are not fixed *a priori*, interannual comparisons cannot be reduced to a simple transfer of class labels between time periods. The purpose of the present study was deliberately narrower. The analysis was designed to document and interpret the sequence of land-cover transformations associated with the catastrophe itself during 2023, including the immediate pre-disaster conditions and the subsequent acute reorganisation of floodplain habitats. In this respect, the study should be regarded primarily as an event-focused reconstruction of post-catastrophic dynamics rather than as a comprehensive long-term anomaly-detection framework.

These limitations also define the main prospects for further research. A priority is the development of methods for interannual matching of unsupervised cluster structures, which would enable the incorporation of multi-year pre-event baselines and a clearer separation of phenological variability from catastrophe-driven change. Future work should therefore extend the current approach to longer time series and broader temporal reference frames, combining unsupervised temporal matching with ecological interpretation of cluster trajectories. Such developments would facilitate a shift from event documentation towards a more general framework for the long-term monitoring of post-catastrophic floodplain transformation.

## Conclusion

Assessing the dynamics of catastrophic landscape change involves specific challenges. Land-cover classification provides qualitative certainty, which is essential for monitoring, management, and the development of strategies for site restoration and biodiversity conservation. Ecological disasters give rise to highly dynamic new habitat types whose occurrence and characteristics are unpredictable, limiting the effectiveness of supervised classification methods. Unsupervised classification requires careful determination of the optimal number of clusters to avoid errors caused by excessive detail. A comprehensive assessment of the spectral characteristics of an area can be achieved through sequential principal component analysis of a broad set of spectral indices. Secondary principal components are sensitive to moisture conditions, soil properties, and indicators of plant physiological status, enabling more detailed and well-founded differentiation of landscape cover.

## Supplemental Information

10.7717/peerj.21869/supp-1 Supplemental Information 1 Raw data: metadata for Sentinel-2 MSI Level-2A scenes. Metadata for all Sentinel-2 MSI Level-2A scenes used in this study to calculate spectral indices and support the spatiotemporal analyses, including acquisition dates, scene folders, and year of acquisition.

10.7717/peerj.21869/supp-2 Supplemental Information 2 Raw data: metadata for shared random sampling points. Metadata for the shared random sampling points used across all Sentinel-2 scenes in the study. includes point identifiers, raster cell identifiers, and spatial coordinates for each sampled analytical unit.

10.7717/peerj.21869/supp-3 Supplemental Information 3 Raw data: temporal trajectories of cluster membership for the sampled analytical units across all analysed dates. The assigned cluster code and cluster name for each sampled point at each date, together with the number of cluster transitions through time.

10.7717/peerj.21869/supp-4 Supplemental Information 4 Derived data: scaling parameters used for pooled z-transformation of spectral indices. Centering and scaling parameters used to standardise spectral indices across all analysed scenes before clustering and related statistical analyses. includes the mean and standard deviation values applied to each spectral index.

10.7717/peerj.21869/supp-5 Supplemental Information 5 Raw data: cluster-level signatures calculated from the spectral index values of the sampled analytical units. Cluster-level summary values for all spectral indices used in the study and serves as a reference for cluster interpretation and comparison.

## Additional Information and Declarations

### Data Availability

The following information was supplied regarding data availability:

The raw data are available in the Supplemental Files.

The data is available at Zenodo: Tutova, H., Lisovets, O., Kunakh, O., & Zhukov, O. (2026). Most informative spectral indices in terms of discriminatory power for cluster differentiation [Dataset]. Zenodo. https://doi.org/10.5281/zenodo.18851861.
